# A lecithinized superoxide dismutase (PC-SOD) improves ulcerative colitis

**DOI:** 10.1111/j.1463-1318.2008.01487.x

**Published:** 2008-11

**Authors:** Y Suzuki, T Matsumoto, S Okamoto, T Hibi

**Affiliations:** * Toho University Sakura Medical CenterSakura, Japan; † Hyogo College of MedicineHyogo, Japan; ‡ Keio University School of MedicineTokyo, Japan

**Keywords:** Ulcerative colitis, superoxide dismutase, PC-SOD

## Abstract

**Objective:**

To assess the safety and efficacy of lecithinized superoxide dismutase (PC-SOD) in patients with ulcerative colitis (UC).

**Method:**

PC-SOD was injected once daily at doses of 40 mg (*n* = 22) and 80 mg (*n* = 20) for a total treatment period of 4 weeks. Efficacy was assessed by UC-Disease Activity Index (DAI) total score. All side effects were recorded and investigated.

**Results:**

At 4 weeks, the UC-DAI total score was significantly decreased *vs* baseline in both the 40 mg and 80 mg groups. It was confirmed that PC-SOD 80 mg was, at least, not significantly superior to PC-SOD 40 mg. Twenty incidences of side effects were noted in 12 (54.55%) of 22 patients in the 40 mg group, while there were three incidences of side effects in two (10.00%) of 20 patients in the 80 mg group. None of these side effects was severe. Thus it was concluded that the test drug is safe when given at daily dosages of 40 mg and 80 mg.

**Conclusion:**

In this pilot study PC-SOD improved UC more rapidly than previously existing drugs. A double blind, placebo-controlled clinical trial of PC-SOD 40 mg/day is required to confirm the efficacy of this agent against UC.

## Introduction

Although the pathogenesis of IBD is not fully understood, it has been well documented that oxidative stress caused by reactive oxygen species (oxygen radicals; ROS) may trigger the onset of IBD along with contributions from immune dysfunction and genetic and environmental factors [1].

Superoxide dismutase (SOD), an enzyme that catalyses the degradation of ROS to oxygen and hydrogen peroxide, was discovered approximately 40 years ago. SOD prevents oxidation in cells of many tissues such as vascular endothelial cells exposed to ROS. SOD is presumably the most potent anti-ROS and was early thought potentially a dream drug. However, as SOD does not bind to cellular membranes and is rapidly excreted from the kidney, it has not been practically used so far [2]. In this regard, several attempts have been made to prepare practical SOD formulations. Although hybrid chimeric SOD (SOD2/3) and recombinant human SOD (h-SOD) improved pharmacokinetics profiles and ameliorated symptoms in animal studies [3], to date h-SOD has not shown beneficial effects in patients [4].

PC-SOD is a lecithinized SOD in which four phosphatidylcholine (PC)-derivative molecules are covalently bound to each SOD dimer. PC-SOD has a high affinity to cellular membranes [5] and efficiently scavenges superoxide anion (O^2−^) produced at affected tissues [6]. Furthermore, PC-SOD has a long half-life in serum [7], maintains enzymatic activity for 6 h after injection [8], and exhibited beneficial effects in animal models of various diseases in which ROS involvement is implicated [7–12].

Thus PC-SOD might be expected to elicit beneficial effects in clinical studies against ROS-mediated diseases such as inflammatory bowel disease and the like.

## Method

Prior to participation in the study, we obtained agreement and received written informed consent from all patients in accordance with the International Conference on Harmonisation General Code of Practice Guidelines.

Subjects enrolled in the present clinical trial were patients who were diagnosed as having UC based on American College of Gastroenterology ‘Ulcerative colitis practice guideline in adults’ [13]. The test drug used was a preparation for intravenous injection containing 40 mg of PC-SOD in one vial (freeze-dried preparation). The test drug was intravenously injected at a dose of 40 or 80 mg by drip infusion for approximately 60 min. The drug was injected once daily initially for 14 days as inpatient treatment followed by twice weekly for another 2 weeks as outpatient treatment. Previously prescribed immunosuppressants (azathioprine, mercaptopurine), other anti-UC agents (mesalazine, salazosulfapyridine), and steroid preparations were concomitantly allowed without any changes of their doses and administration routes and schedules. However, concomitant cyclosporine and centrifugal leukocyte aphaeresis therapy were prohibited. Although all participants were followed until endoscopy was carried out at 1 month following injection, in this study follow-up could not be continued thereafter because other therapies were subsequently given to most patients.

Efficacy assessment of the test drug was conducted based on four items (stool frequency, bloody stool, mucosal findings, and doctors’ global assessment) in Ulcerative Colitis-Disease Activity Index (UC-DAI) [14]. The UC-DAI total score was calculated at baseline and week 4 or at the withdrawal visit for patients who early discontinued. Patients whose UC-DAI total score at 4 weeks was ≥ 2 points lower than the baseline level were defined as ‘improved.’

Safety assessment of the test drug was conducted based on incidence rates of side effects and abnormal clinical parameters at baseline and at 1, 2, and 4 weeks of treatment.

## Results

### Baseline characteristics

Of 42 patients enrolled, 22 and 20 patients were randomly assigned to receive PC-SOD 40 and 80 mg, respectively. The mean disease duration (± SD) was 59.1 ± 48.0 and 78.8 ± 74.5 months, respectively. Baseline characteristics such as age, body weight, sex, and disease duration were not significantly different in the two groups. In all, three patients who withdrew from the study were excluded from the efficacy assessment — one in the 40 mg group and two in the 80 mg group.

### Efficacy

**Figure 1:**
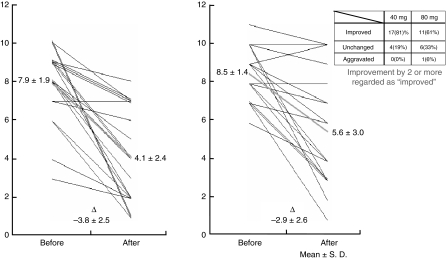
Improving effects of PC-SOD on UC-DAI total score in patients with active ulcerative colitis. UC-DAI total score in individual subjects at baseline and after treatment with PC-SOD 40 mg (left graph) and 80 mg (right) is connected by lines. Ordinate: UC-DAI total score. Note that the UC-DAI total score in most patients was lowered after treatment. Mean (SD) UC-DAI total score in the 40 mg group at baseline (7.9 ± 1.9) was significantly reduced by 3.8 ± 2.5 and that in the 80 mg group (8.5 ± 1.4) significantly lowered by 2.9 ± 2.6. The inserted table indicates the rate of patients improved, unchanged, and aggravated following treatment with PC-SOD. ‘Improved’ was defined as subjects whose UC-DAI total score after treatment was reduced by ≥ 2 points.

As shown in Fig. 1, UC-DAI total score in most patients was reduced in both the 40 mg and 80 mg groups at 4 weeks *vs* baseline. ‘Improved’ patients numbered 17 (81%) and 11 (61%) in the 40 mg and 80 mg groups, respectively (inserted table in Fig. 1). Mean (SD) UC-DAI total scores at 4 weeks in the 40 mg (4.1 ± 2.4) and 80 mg (5.6 ± 3.0) groups were significantly lower than their average baseline levels (40 mg group, 7.9 ± 1.9; 80 mg group, 8.5 ± 1.4), indicating that PC-SOD improved UC.

### Safety

Among the 42 patients, there were 23 incidences in 14 patients (33.3%) of side effects possibly related to taking the test drug. In the 40 mg group there were 20 patients of side effects appearing in 12 (54.5%)of 22 patients assessed, while in the 80 mg group three patients of side effects were recorded in two (10.0%)of 20 patients assessed. Side effects included nausea (2.4%), fever (2.4%), headache (2.4%), malaise (2.4%), and low back pain (2.4%). None of the side effects was severe and the incidence of side effects was not related to the dose of PC-SOD. Abnormal laboratory tests included increased WBC (7.1%), elevated γ-GTP (4.8%), and positive urinary protein (4.8%).

## Discussion

Reactive oxygen species are produced as a by-product of normal oxygen metabolism and degraded by defense mechanisms including SOD. Overproduction of ROS exceeding the degradation capacity of such defense mechanisms is implicated in the pathogenesis of inflammatory diseases such as IBD, neurodegenerative diseases, cardiovascular diseases, and cancer. Although SOD is anti-oxidant, overexpression of this enzyme conversely produces ROS. Thus a balance between ROS and SOD is important [2].

In this study PC-SOD given at a daily dosage of 40 and 80 mg significantly lowered UC-DAI total score, the major assessment item in UC, at 4 weeks *vs* baseline. There was no significant difference between the two treatment groups. Furthermore, ‘noninferiority’ analysis confirmed that the 80 mg group was, at least, not significantly superior to the 40 mg group (data not shown). Recently, PC-SOD was reported to activate SOD activity linearly in men at doses from 20 to 80 mg in a clinical study to investigate pharmacokinetics as well as safety and tolerability of PC-SOD in men. Particularly, PC-SOD increased SOD activity over baseline levels from 8 to 19 h at doses of 40 and 80 mg respectively, that is at the same doses as were used in the present study [15]. Thus beneficial effects of PC-SOD against IBD were strongly suggested by improvement of half-life and affinity to the cell membrane [5,6].

In this study follow-up could not be continued after 1 month because other therapies were subsequently given to most patients. Furthermore, their therapies were not restricted. Thus although a question of interest it could not be determined whether remissions occurred in subjects following discontinuation of test therapy.

Although the incidence of side effects was higher in the 40 mg group than in the 80 mg group, none of the side effects was severe. As efficacy was similar in the two groups and side effects were not dose related, the optimum dose of the test drug was concluded 40 mg.

Although efficacy of the test drug was assessed at 4 weeks in the present study, many previously existing drugs were clinically tried over an 8-week period. As PC-SOD elicited significant improvements of UC at 4 weeks in the present study, the beneficial effects of PC-SOD might appear earlier than those of other drugs such as mesalazine and the like. A double blind, placebo-controlled clinical trial of PC-SOD 40 mg/day is required to confirm the efficacy of PC-SOD against UC.
